# Application of *Corynebacterium glutamicum* engineering display system in three generations of biorefinery

**DOI:** 10.1186/s12934-022-01741-4

**Published:** 2022-01-28

**Authors:** Kerui Lin, Shuangyan Han, Suiping Zheng

**Affiliations:** 1 Guangdong Key Laboratory of Fermentation and Enzyme Engineering, School of Biology and Biological Engineering, South China University of Technology, Guangzhou, 510006 People’s Republic of China; 2 Guangdong Research Center of Industrial Enzyme and Green Manufacturing Technology, School of Biology and Biological Engineering, South China University of Technology, Guangzhou, 510006 People’s Republic of China

**Keywords:** *Corynebacterium glutamicum*, Surface display system, Biorefinery, Transport, Metabolic network reconstruction

## Abstract

The fermentation production of platform chemicals in biorefineries is a sustainable alternative to the current petroleum refining process. The natural advantages of *Corynebacterium glutamicum* in carbon metabolism have led to *C. glutamicum* being used as a microbial cell factory that can use various biomass to produce value-added platform chemicals and polymers. In this review, we discussed the use of *C. glutamicum* surface display engineering bacteria in the three generations of biorefinery resources, and analyzed the *C. glutamicum* engineering display system in degradation, transport, and metabolic network reconstruction models. These engineering modifications show that the *C. glutamicum* engineering display system has great potential to become a cell refining factory based on sustainable biomass, and further optimizes the inherent properties of *C. glutamicum* as a whole-cell biocatalyst. This review will also provide a reference for the direction of future engineering transformation.

## Introduction

Current industrial chemical products mainly use fossil energy including petroleum, coal, and natural gas as raw materials, and account for about 85% of energy use [1, 2]. Fossil resources are not sustainable, and their combustion by-product, carbon dioxide, is the main cause of global warming, accounting for approximately 52% of global warming factors [3]. The conversion to alternative energy sources from the extraction of fossil fuels leads to a more sustainable economy with renewable resources and has attracted worldwide attention. The utilization of biomass resources provides possibilities for the integrated production of chemistry, materials, energy, and food [4]. The concept of bio-refining emerged and gradually evolved. The biorefining concept is similar to that of traditional petroleum refining and uses biomass as a raw material to obtain a series of products, including commodities, and fine and specialty chemicals [5]. The International Energy Agency (IEA) defines bio-refining as "'the sustainable processing of biomass into a spectrum of marketable products and energy" [6]. Biorefineries have different definitions and classifications according to different conditions, platform technologies, final products, raw materials, and conversion processes. The biorefineries introduced in this review are classified according to the source of the raw materials. Three different types of bio-refineries can be used, depending on whether the raw materials used as raw materials come from starch and other grains (first-generation biorefinery), agricultural waste (second-generation biorefinery), or CO2 or toxic environmental pollution (third-generation biorefinery) [5, 7–10].In addition, industrial biorefineries have been identified as the most promising route to the creation of a new domestic bio-based industry [11].

At present, a variety of compounds and commodities are produced through biorefinery. Industrial microorganisms such as *Escherichia coli*, *Saccharomyces cerevisiae*, and *Corynebacterium glutamicum* are designed as microbial cell factories that can use biomass to produce value-added platform chemicals and polymers. Compared with *E. coli* and *S. cerevisiae* [12], *C. glutamicum* has shown great potential to surpass other industrial microorganisms and has many advantages. First, *C. glutamicum* has low extracellular protease activity and can secrete properly folded functional precursor proteins [13]. Second, *C. glutamicum* has a weak carbon catabolite inhibitory effect and can use mixed sugars as a carbon source without significant growth retardation [14, 15]. Third, *C. glutamicum* is robust and exhibits tolerance to organic acids, furfural, and toxic aromatic compounds. Moreover, *C. glutamicum* cells maintain a strong catalytic function under growth-inhibiting conditions and maintain high cell growth with high-density fermentation [16–18]. These features are of great significance to the toxic substances produced during lignocellulose treatment and environmental pollutant degradation. Fourth, *C. glutamicum* has a wide spectrum of natural carbon source substrates. It has a natural metabolic network for five-carbon sugars (D-xylose and L-arabinose), six-carbon sugars (glucose, and mannose), monosaccharides (maltose), and toxic aromatic compounds [19, 20]. The spectrum of *C. glutamicum* substrates involves three generations of bio-refining raw materials. Additionally, *C. glutamicum* is safe, has a clear genetic manipulation background, can be used to produce a variety of compounds, and can convert cheap biomass into high-value products.

**Fig. 1:**
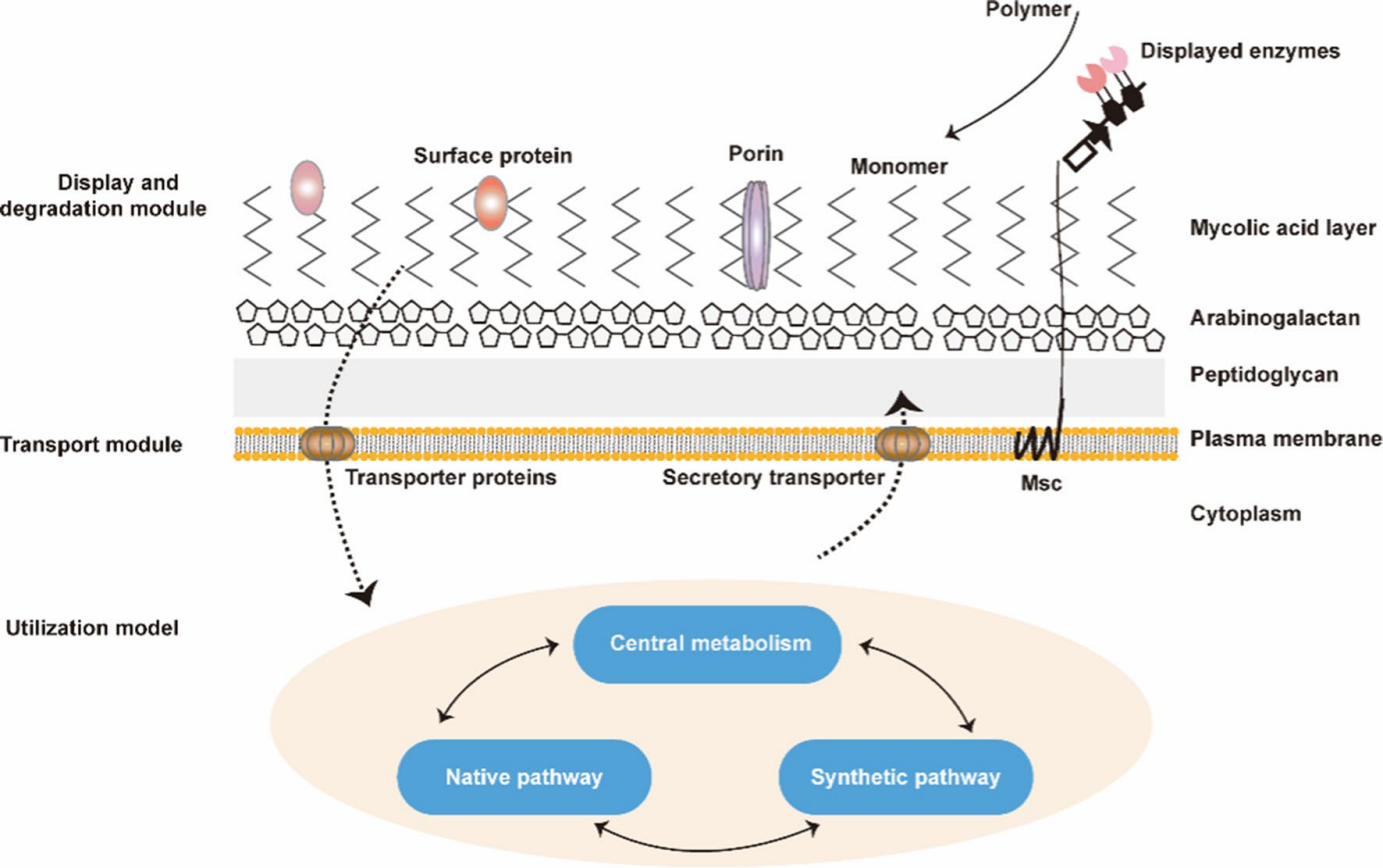
Engineering transformation of the *C. glutamicum* system for the display and degradation, transport, and metabolic network reconstruction models. Display and degradation modules include anchored protein and polymer lyase; the transport module includes ATP-dependent, ion channels, PTS, secondary transporters and artificial transporters; the utilization module (metabolic network reconstruction models) include central metabolic pathways, natural metabolic pathways, and artificial metabolic pathways

The direct application of *C. glutamicum* in biorefineries is limited because it cannot secrete effective hydrolytic enzymes to utilize the biorefinery raw materials. To address this limitation, *C. glutamicum* has been engineered to optimize and construct the degradation module (consisting of the anchor protein and display enzyme), the transport module (pentose, hexose, and other transporters), and utilization module (natural metabolic network and artificial metabolic network) to perform biological treatment of biomass raw materials and compound production by expressing and displaying various functional genes (Fig. 1). These engineering technologies have expanded the *C. glutamicum* substrate spectrum, enhanced its utilization of renewable biomass resources, and have provided important incremental improvements to the economics of industrial-scale manufacturing (Fig. 1). *C. glutamicum* will use renewable resources to produce natural or non-natural products, making it a biorefinery cell factory with economic benefits and prospects.

### *C. glutamicum* and its engineering display system

*C. glutamicum* is a fast-growing, aerobic, and non-pathogenic Gram-positive soil bacterium that is a generally considered safe (GRAS) industrial microorganism. *C. glutamicum* has been studied for over 50 years [21]. In the food and feed industries, *C. glutamicum* is the main production strain of amino acids such as glutamic acid and lysine [22, 23], and it has been engineered to produce industrial and commercial chemicals. For example, biofuels (such as ethanol, isobutanol, and higher alcohols), organic acids (such as lactic acid, pyruvic acid, succinic acid, glutaric acid, γ-aminobutyric acid, methyl salicylic acid, and 5-aminovaleric acid [24–26]), and monomers or precursors for biopolymers (including lactate, succinate, putrescine (1,4-Diaminobutane), cadaverine (1,5-diaminopentane), adipic acid, terephthalic acid, 1,2-propanediol, 4-hydroxybenzoic acid (4-HBA), and 1-(2, 4,6-trihydroxyphenyl)butane-1,3-dione (TPBD) [27–31]) and other high value-added compounds such as resveratrol [16, 32–34]. Additionally, the lack of endotoxin and low extracellular protease content, make *C. glutamicum* a potential host for the production of recombinant proteins, including pharmaceutical proteins (single-chain Fv [35, 36] and Fab fragment [37]) and industrial enzymes (amylase [38–42], glycosidase [43, 44], etc.). Metabolic engineers have been exploring alternative renewable carbon sources that have competitive value in the food and feed industries for the production of commercial chemicals of *C. glutamicum *(Table 1).

However, surface display technology gives *C. glutamicum* the ability to utilize renewable carbon sources because the display of functional proteins on the cell surface gives host cells new functions to produce chemicals from cheap biomass [45]. Compared with secreted enzymes, the displayed enzymes have many advantages, including improved stability of enzyme activity [46, 47] and enzyme thermal stability [48], convenient recovery [49] and wide application value (whole cell catalysis, combinatorial library screening, protein engineering, bioremediation, biosensor, biofuels production, etc.) [50–53]. To display proteins on the surface of *C. glutamicum*, several anchoring motifs have been used. At present, the display system of *C. glutamicum* only uses three types of anchoring proteins: foreign proteins PgsA, mycoloylated proteins, and membrane proteins. PgsA is a transmembrane protein derived from *Bacillus subtilis* and is part of the poly-γ-glutamate synthase complex [42]. Mycoloylated proteins are special proteins that exist on the mycolic acid layer of the *C. glutamicum* outer membrane and are fixed on mycolic acids through O-acylation covalent modification, which occurs on serine residues [54]. The currently known mycoloylated proteins that can be used as anchor motifs are NCgl1337, ion-selective channel protein NCgl0933 (porin B, PorB), NCgl0932 (porin C, PorC), and PorH(NCgl number of PorH have not been assigned [55]) [39, 40]. PorH combines with PorA to form a cation-selective channel called the PorHA channel. In the absence of PorHA channels, a functional anion-selective channel, PorB, is necessary for optimal growth [56]. Among the *C. glutamicum* membrane proteins, only NCgl1221 (mechanosensitive channel, MscCG) has been developed as an anchor protein[41]. NCgl1221 is also the main glutamate efflux system of *C. glutamicum* [57]. Recombinant *C. glutamicum*, with these anchored proteins, has been modified to display carbohydrate-active enzymes (CAZy) to expand the carbon sources available during fermentation and to enable *C. glutamicum* to ferment cheap raw materials into valuable biological products. These CAZy include amylase [38–42], glucanase [58], glycosidase [43, 44], and cellulase complex [59].

**Fig. 2:**
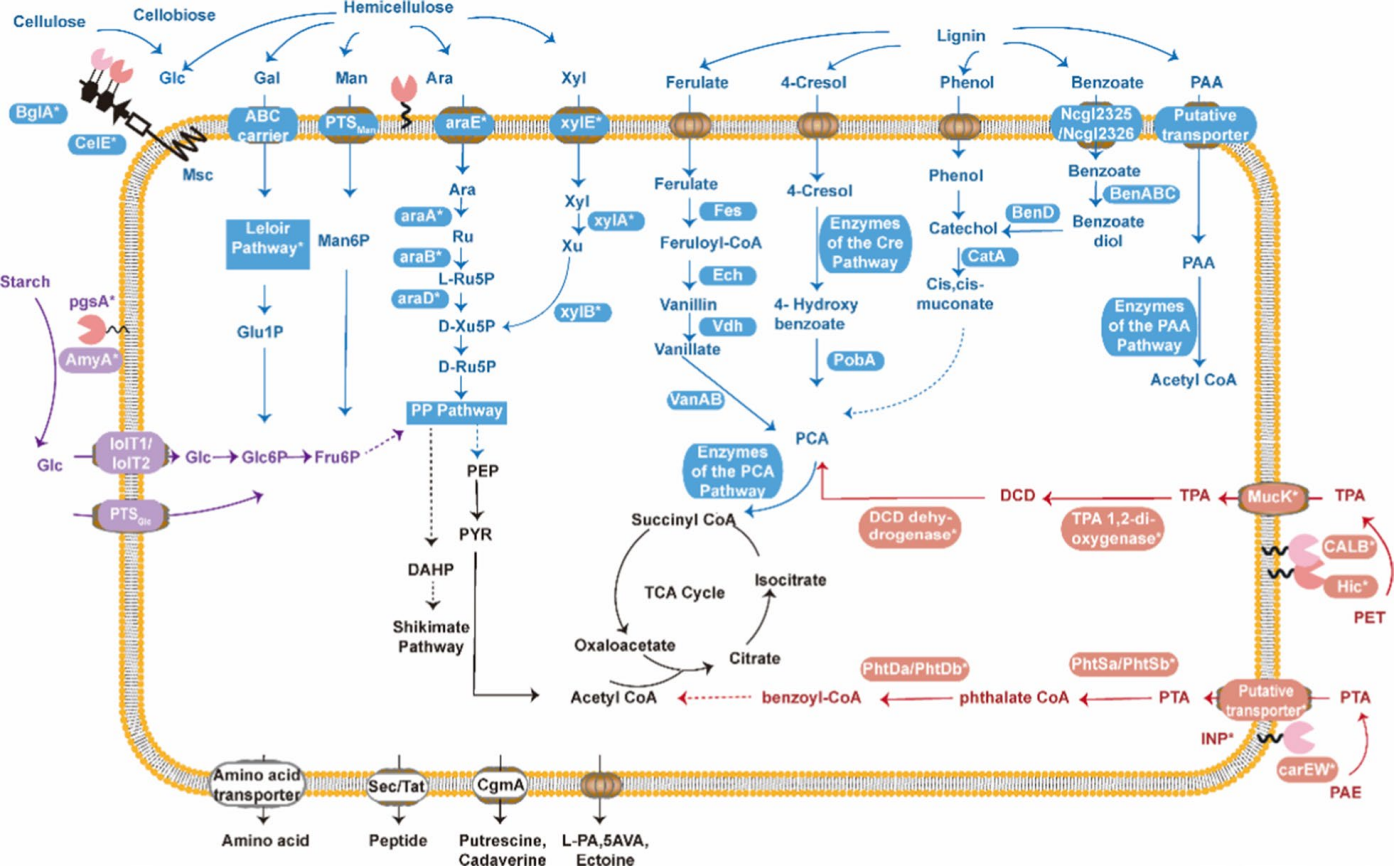
Utilization of three generations of biorefinery materials based on the surface display system of *C. glutamicum*, including surface display, transporters, and metabolic reactions. Overview of the surface display, transporters, and metabolic reactions in *C. glutamicum* for uptake and conversion of three generations of biorefinery materials. Black represents the endogenous reaction of *C. glutamicum*, and * represents foreign proteins expressed by heterologous genes. Purple indicates the utilization path of the first-generation of biorefinery, blue indicates the utilization path of the second-generation of biorefinery, and orange indicates the possible utilization path of the third-generation of biorefinery. The solid line represents direct reactions, and the dashed line represents indirect reactions. Enzymes of the Cre pathway: CreHI, CreJEF, CreG, CreC, and CreD; enzymes of the Paa pathway: PaaK, PaaABCDE, PaaG, PaaZ, PaaG/J, PaaF, PaaH, and PaaJ; amino acid transporter: LyaE for L-lysine, L-arginine, L-citrulline; CgmA for L-arginine; YggB: L-glutamate; ThrE for L-threonine, L-serine; BrnEF: L-valine, L-leucine, L-isoleucine, L-methionine. *Glc* glucose, *Glc6P* glucose-6-phosphate, *Fru6P* frucose-6-phosphate, *Xyl* xylose, Ara, arabinose, *CelE* endoglucanase E, *BglA* β-glucosidase A, *Msc* mechanosensitive channel, Glc1P, glucose-1-phosphate, *F6P* fructose-6-phosphate, *PYR* pyruvate, *Gluc6P,*gluconate-6-phosphate, *Glc6P* gluconate-1-phosphate, Man, mannose, *Man6P* mannose-6-phosphate, *Ara* arabinose, *AraA* arabinose isomerase, *AraB* ribulokinase, *AraD* ribulose 5-phosphate 4-epimerase, *Ru* ribulose, *L-Ru5P* L-ribulose-5-phosphate, *D-Ru5P* D-ribulose-5-phosphate, *PEP* phosphoenolpyruvate, *DHAP* 1,3-dihydroxyacetone phosphate, and *PCA* protocatechuate. *PTS* phosphoenolpyruvate phosphotransferase, *Xylu5P* xylulose-5-phosphate, *D-Ribo5P* ribose-5-phosphate, *Xyl* xylose, *XylA* xylose isomerase, *XylB* xylulokinase, *Xu* xylulose, *PP* pathway, pentose phosphate pathway, *TCA* tricarboxylic acid cycle, *Vdh* putative vanillin dehydrogenase gene, *vanAB* genes encoding subunits A and B of vanillate demethylase, *PAA* phenylacetic acid, *Fcs* feruloyl-CoA synthetase, *Ech* enoyl-CoA hydratase/aldolase, *PobA* 4-hydroxybenzoate 3-hydroxylase (NCgl1032), *CatA* catechol 1,2-dioxygenase (NCgl2319), *BenABC* benzoate dioxygenase complex, encoded putatively by NCgl2320, NCgl2321, and NCgl2322, *benD* 2-hydro-1,2dihydroxybenzoate dehydrogenase (putatively encoded by NCgl2323), *MucK* a native MFS transporter known to import muconate in ADP1 (from *Acinetobacter baylyi*), *DCD* 1,2-dihydroxy-3,5-cyclo-hexadiene-1,4-dicarboxylate

*C. glutamicum* uptake of some of the display system degradation products requires the transport system. The transmembrane transport system in *C. glutamicum* includes ATP-dependent ion channels, the phosphotransferase system (PTS), and secondary transporters [29, 60, 61]. The transport of many hexoses is involved in the phosphorylation cycle of the PTS system [62]. Pts are composed of two common energy-coupling cytoplasmic proteins, enzyme I (EI) and histidine-containing phosphor carrier protein (HPr), which are encoded by ptsI (NCgl1858) and ptsH (NCgl1862), respectively, and a series of sugar-specific enzyme II complexes (EII) [63–65]. Currently, four sets of PTSs have been identified in *C. glutamicum*, each of these PTSs is specific for a different substrate and those identified are specific for glucose, fructose, sucrose, and an unknown substrate [66]. In addition, *C. glutamicum* can also transport glucose through inositol transporters coded by iolT1 and iolT2 [67]. For aromatic compound transport, most of the functionally identified aromatic compound transport systems belong to either the aromatic acid/H + symporter (AAHS) family of transporters within the major facilitator superfamily (MFS) or the ATP-binding cassette (ABC) superfamily [68]. In *C. glutamicum*, two benzoate transporters are encoded by NCgl2325 and NCgl2326. Additionally, a gene encoding a putative phenylacetate transporter (CgR_0643, NCgl numbers have not been assigned) was observed in the putative paa cluster of *C. glutamicum* strain R [69]. With the expansion of the substrates of *C. glutamicum*, artificial transport systems for various substrates have been developed through gene editing (Fig. 2), and a series of methods have been developed for *C. glutamicum* through reconstruction and optimization of metabolic pathways. In addition, through restructuring and optimizing metabolic pathways, a series of methods have been developed for the uptake, transport, metabolism, and production of the biomass carbon source in *C. glutamicum*. In the next section, this review will introduce the use of engineering *C. glutamicum* based on the surface display system in the three generations biorefining raw materials and the engineering transformation in this process (Fig. 2).

### Application of *C. glutamicum* and its surface display system in the first-generations of biological refining

The first generation of biorefinery uses starch and other grains as raw materials to produce biofuels and chemicals. Starch is used as the main carbon source for the industrial production of amino acids, but *C. glutamicum* cannot directly utilize starch [70]. In the past ten years, researchers have realized the direct utilization of starch and product production by *C. glutamicum* by displaying amylase on the surface. Tateno et al. showed that α-amylase (from *Streptococcus bovis*) surface displayed by pgsA produced 6.04 g/L lysine [42], 15.5 ± 1.3 g/L glutamic acid [41], poly-β-hydroxybutyrate (PHB) [71], cadaverine [72], 88.9 g/L lactic acid, and 14.0 g/L succinic acid [73] (Fig. 2). In addition, mycolic acid layer proteins PorC, NCgl1337, and membrane protein Msc were used as anchoring motifs to display α-amylase, and these recombinant strains produced glutamic acid or L-lysine from starch successfully [39, 41, 44] (Table 1).

**Table 1:** Utilization of three generations of biorefinery raw materials based on the surface display system of *C. glutamicum*

	Substrate	Anchorprotein	Passengerprotein	Resource of passenger protein	Producer	Product	Titer(g/Lmedium)	References
First generation of biorefinery	Starch	PgsA	α-amylase(AmyA)	*Streptococcusbovis148*	*ATCC13032*	L-Lysine	6.04	Tateno et al. [42]
		PgsA	α-amylase(AmyA)	*Streptococcusbovis148*	*ATCC13032*	Polyhydroxybutyrate(PHB)	6.4wt%	Song et al. [47]
		PgsA	α-amylase(AmyA)	*Streptococcusbovis148*	*ATCC13032*	Organic Acids	107.8	Tsuge et al. [73]
		NCgl1221	α-amylase(AmyA)	*Streptococcusbovis148*	*ATCC13869*	L-glutamate	19.3	Yao et al. [41]
		NCgl1337S	α-amylase(AmyA)	*Streptococcusbovis148*	*ATCC13032*	L-Lysine	4.39	Choi et al. [39]
		NCgl1337	α-amylase(AmyA)	*Streptomyces coelicolor A3(2)*	*ATCC13032*	L-Lysine	1.75	Choi et al. [39]
Second generation of biorefinery	Xylooligosaccharides	PorH	β-xylosidase(Xyl)	*Bacillussubtilis*	*PIS8*	1,5-Diaminopentane(cadaverine)	1.28	Imao et al. [127]
	Hemicellulose	NCgl1337/ NCgl1337S	Endoxylanase(XlnA)	*Streptomyces coelicolor A3(2)*	*ATCC13032*	Xylooligomers	-	Choi et al. [39]
	Cellobiose	Porin(porC)	β-glucosidase(Sde1394)	*Saccharophagusdegradans*	*ATCC13032*	L-Lysine	1.08	Adachi et al. [44]
		Porin(porC)	β-glucosidase(Sde1394)	*Saccharophagusdegradans*	*DM1729*	L-Lysine	0.73	Anusree et al. [58]
Third generation of biorefinery	CO_2_	Porin(porB)	carbonic anhydrase (CA)	*Neisseria gonorrhoeae*	*C. glutamicum*	Bicarbonates	-	Koo et al. [111]

### Application of *C. glutamicum* and its surface display system in the second-generation of biorefinery

The first-generation raw material has adverse effects on food prices and the environment [74, 75]. However, lignocellulose, the main raw material of the second-generation biorefinery, is a complex heteropolymer composed of cellulose, hemicellulose, and lignin that is formed through hydrogen and covalent bonds. It is a complex heteropolymer that can be used to produce biofuels and abundant renewable raw materials for commercial chemicals [76]. Lignocellulose is composed of 40–50% cellulose, 25–35% hemicellulose, and 10–20% lignin [77]. Cellulose is composed of cellobiose, which is a homopolymer of glucose. Hemicellulose is composed of various hexoses (D-glucose, D-mannose, and D-galactose) and pentoses (D-xylose Xyl and L-Arabinose Ara). The total content of pentose sugars in lignocellulose hydrolysates reaches 5–20% xylose, 1–5% arabinose, and 10%–20% mannose [78, 79]. Currently, these monomers are utilized in *C. glutamicum* through natural or engineered metabolic networks. Utilizing the natural ability of *C. glutamicum* to degrade and utilize different sugars, establish effective sugar degradation and transport pathways. This further improves the conversion efficiency of renewable resources and avoids competition between biorefineries and food or feed supplies.

### Cellulose

Cellobiose is a disaccharide composed of two glucose monomers connected with β-1-4-glycosidic bonds. The degradation of cellulose is generally carried out by a combination of a variety of cellulase enzymes, such as endoβ-1,4-glucanase (EC 3.2.1.4), exo-β-1,4-glucanase, cellobiohydrolase (EC 3.2.1.91), or β-glucosidase (BGL) (EC 3.2.1.21). Kim used Msc to display the cellulase complex endoglucanase E (CelE) and β-glucosidase A (BglA) enzymes on the C. glutamicum cell surface to completely degrade cellulose into glucose. The synergistic effect of these two cellulases significantly improved the saccharification efficiency and thermal stability. The activity of the displayed cellulase complex was higher than that of the secreted cellulase complex [59]. Adachi used porins to display Sde1394 (a BGL from Saccharophagus degradans) on the surface of C. glutamicum cells and to directly assimilate cellobiose as a carbon source. The recombinant strain produced 1.08 g/L of L-lysine from 20 g/L of cellobiose, which was approximately three times higher than the L-lysine produced by secreted BGL in C. glutamicum [44]. In addition, by co-expressing these CAZy in C. glutamicum, glucose, xylose, and arabinose produced by lignocellulose can be used together, and the products produced do not inhibit enzyme activity. The combined utilization of these polysaccharides is of great significance for the practical industrial application of lignocellulose [58, 80, 81]. Shortly, this surface display of multiple enzymes on a single cell will greatly promote whole-cell catalysis [49]. The combined utilization of these polysaccharides is of great significance for the practical industrial application of lignocellulose [58, 81].

### Hemicellulose

The utilization of hemicellulose by C. glutamicum mainly focuses on the transport of hemicellulose hydrolysates, and there are few studies on display involved. Hydrolyzed hemicellulose produces hexoses (D-glucose, D-mannose, and D-galactose) which are transported into the cell through the PTS transport system [29] or the ABC carrier [82] (Fig. 2). Glucose is transported by PTS and produces Glc6P or is transported by lolT1/lolT2 and then converted to Glc6P by hexokinase before entering the glycolytic pathway. The mannose 6-phosphate produced by PTS transport of mannose is reversibly converted to fructose 6-phosphate by the phosphate isomerase(*Pmi*) before entering glycolysis. As for galactose, wild-type *C. glutamicum* lacks the galactose metabolism network. The engineered *C. glutamicum* containing *L. lactis subsp. cremoris* MG1363 galactose operon, including aldose-1-epimerase (*galM*), galactokinase (*galK*), udp-glucose-1-p-Glutamate strains with uridyltransferase (*galT*), and UDP-galactose-4-epimerase (*galE*) genes, can grow and produce lysine with galactose as the sole carbon source [83].

At present, the research on the utilization of the hemicellulose products of *C. glutamicum* is mainly xylose and arabinose. Most wild-type *C. glutamicum* lack effective xylose and arabinose transport or metabolic pathways. Currently, three transmembrane proteins that transport xylose have been used in *C. glutamicum*: lolT1 [84], XylE [85], and AraE [86]. Most studies on *C. glutamicum* Xylose metabolism have focused on the xylose isomerase (XI) pathway and the Weimberg (WMB) pathway. Researchers have expressed that *xylA* (xylose isomerase) and *xylB* (xylose kinase) (from *E. coli* and *Xanthomonas campestris*) can utilize D-xylose and produce L-ornithine, lactic acid, and succinic acid in *C. glutamicum* [87, 88]. Imao et al. used PorH ankyrin to display β-xylosidase (from *B. subtilis*) on the cell surface of *C. glutamicum*, and expressed xylAB (from *E. coli*). The engineered strain could produce 12.4 mM lysine and 11.6 mM 1,5-diaminopentane from 11.9 g/L xylo-oligosaccharides. Jin et al. used engineered *C. glutamicum* GJ04 to produce 61.7 g/L glutamic acid from xylose and glucose derived from lignocellulose. Additionally, most wild-type C. glutamicum lack the arabinose transport pathway as well, and a few species of C. glutamicum, such as C. glutamicum ATCC 31831, have the endogenous AraE arabinose transporter [19]. AraE is a membrane channel protein with xylose transport function, which can simultaneously transport L-arabinose. Arabinose is converted into D-xylose-5-phosphate by the action of arabinose isomerase (araA), ribonucleic acid kinase (araB), and ribulose-5-phosphate 4-epimerase (araD) to enter the PP pathway. However, because hydrolyzed cellulose is composed of glucose, xylose, arabinose, and other sugars, it can be considered that the fermentation of mixed sugars is the main bottleneck for its utilization [89].

### Lignin

Lignin is composed of coniferyl alcohol, sinapyl alcohol, and p-coumarol benzyl alcohol units, and can be used for the production of aromatic-derived fuels and chemicals [90]. Unfortunately, the complex chemical structure of lignin is a raw material that is difficult to depolymerize and degrade. Lignin decomposition products are mainly aromatic compounds, which are usually toxic to microorganisms commonly used in biological production. C. glutamicum can not only tolerate and degrade aromatic hydrocarbons, but can also use many aromatic compounds derived from lignin (including ferulic acid, vanillin, phenol, benzoic acid, phenylacetic acid, and 4-cresol) as the only carbon source for growth and energy [91]. C. glutamicum has a remarkable ability to utilize aromatic compounds, which makes it unique advantages in the use of lignocellulose hydrolysates in industrial fermentation. Becker et al. engineered C. glutamicum to produce Cis, cis-muconic acid (MA) from glucose, catechol, phenol, and benzoic acid. After hydrothermal depolymerization of softwood lignin into small aromatic hydrocarbons, the strain accumulated 1.8 g/L MA from the hydrolysate [92]. Kallscheuer et al. studied the degradation pathway of ferulic acid in C. glutamicum and expressed genes encoding chalcone synthase (CHS) and chalcone isomerase (CHI), and produced naringenin and mustard caffeic acid from phenylalanine p-coumaric acid and phenylalanine. In addition, the research team knocked out 27 genes of C. glutamicum, eliminating most of the catabolism pathways of known aromatic compounds. The resulting strain produces 2-hydroxybenzoate, 3-hydroxybenzoate, protocatechuate, and 4-hydroxybenzoate from intermediates of aromatic amino acids in the shikimate pathway [33, 93, 94].

The high tolerance of C. glutamicum to aromatic compounds may be due to its typical outer membrane-like structure (mycomembrane), which is mainly composed of fungal acids and can act as a permeation barrier to toxic aromatic compounds [95]. However, the high resistance to aromatic compounds and complex catabolic network also prevents the metabolism and production of C. glutamicum during the production of aromatic compounds (except aromatic amino acids). This prevents C. glutamicum from being used as a medicine or being used as a production host of aromatic compounds involved in biotechnology [69, 93]. At present, researchers have realized the utilization of lignin hydrolysate and product production through engineering transformation of C. glutamicum, but few display technologies of C. glutamicum used in the degradation of lignin polymers. This may be due to the limitation caused by the low expression of the foreign protein of C. glutamicum. In this review, we described the metabolism and regulation of C. glutamicum genes involved in the metabolism of lignin-derived aromatic compounds. It is expected that the display technology of *C. glutamicum* is a feasible direction for lignin degradation and utilization (Fig. 2) [18, 96–101]

### Application of *C. glutamicum* and its surface display system in the third-generation of biorefinery

The third-generation biorefinery relies on the use of toxic waste, syngas, microalgae, or bacteria that use sunlight for production. The second-and third-generation bio-refineries will reduce the cost of raw material processing, reduce the demand for resources such as food and water, further promote sustainable clean and green biological manufacturing [5, 7]. Corynebacteria are soil and saprophytic microorganisms with cold-tolerant cell walls and have been used in various bioremediation fields. The *C. glutamicum* has a high tolerance to the polymer units of petroleum pollutants, plastics, and pesticides and possesses a natural metabolic network. There are also potential high-yielding strains of these units. Current research mostly focuses on the utilization of *C. glutamicum* in the decomposition unit of these pollutants, and there is little research about the surface display system. However, the decomposition of polymer pollutants is generally based on chemical decomposition, and it is expected that degradation through demonstration technology is a feasible future direction. Therefore, we mainly discuss progress in the utilization of *C. glutamicum* for environmental pollutants such as petroleum pollutants, plastics, pesticides, and their derivatives, as well as possible surface display solutions. It is expected that *C. glutamicum* is a potential bacterial substrate for use in future bioremediation.

The main components of most petroleum pollutants, plastics, and pesticides are aromatic compounds. *C. glutamicum* can degrade a variety of aromatic monomers. *C. glutamicum* has a metabolic network involving the following aromatic compounds: benzoic acid, phenol, 3-hydroxybenzoic acid, gentisic acid, protocatechuic acid, vanillic acid, p-hydroxybenzoic acid, 4-cresol, resorcinol, benzyl alcohol, 2,4-dihydroxybenzoic acid, 3,5-dihydroxytoluic acid, naphthalene, vanillin, ferulic acid, cinnamic acid, caffeic acid, and coumaric acid [69] (Fig. 2). *C. glutamicum* is highly resistant to various toxic aromatic compounds such as phenol, protocatechuic acid, 4-HBA, and 4-ABA, and this feature of *C. glutamicum* is not observed in other industrial microorganisms including *E. coli* and *B. subtilis*. The high tolerance of *C. glutamicum* to toxic aromatic compounds is a key factor in the production of high-valence aromatic compounds. However, most industrially important aromatic compounds are produced through the chemical conversion of petroleum-based raw materials, such as benzene, toluene, and xylene. Therefore, *C. glutamicum* can connect the degradation of environmental pollutants with the production of commercial plastics, ensuring sustainable green development and avoiding the fossil route [102].

In terms of display technology, researchers have achieved the degradation of aromatic polymers by displaying functional enzymes on the surface of yeast. Chen et al. used GPI-modified cell wall proteins to display PETase(polyethylene terephthalate-degrading enzyme) on the surface of *Pichia pastoris* cells to achieve high-efficiency biodegradation of PET(polyethylene terephthalate) and improve the pH and thermal stability [102]. Ding et al. displayed the carboxylesterase CarEW (from *Bacillus sp. K91*) and GFP on the surface of *E. coli* through ice nucleation protein. They found that 1.5 mg/ml of DiBP (diisobutyl phthalate, one of the main PAE(phthalic acid esters) plasticizers) was degraded by 10 U of surface-displayed CarEW cells in 120 min [103]. In addition, PET and PAE degradation systems, and their monomeric terephthalic acid and phthalic acid transport systems [104–108], and metabolic networks [100, 109] are gradually improving, making it feasible for the application of *C. glutamicum* display system in these polymers. Therefore, this review envisages the possible PET and PAE degradation pathways in the *C. glutamicum* display system (Fig. 2).

Researchers recently proposed the concept of CO_2_ substrate-based third-generation biorefinery for green biomanufacturing [5, 7–10, 110]. *C. glutamicum* is used in a large number of industrial applications due to its large-scale production process and transgenic stability, and it is a feasible research direction in the capture and application of CO2. Koo et al. used porB to display carbonic anhydrase (CA) on the surface of *C. glutamicum* to achieve effective CO2 capture and CO2 hydrate [111]. In addition, Researchers have used combined biological treatment to utilize CO_2_-fixed biomass in engineered *C. glutamicum*. Lee et al. used engineered *C. glutamicum* bacteria and microalgae to jointly process succinic acid production. The engineered strain that secretes amylase can produce succinic acid (0.28 g succinic acid/g total sugars including starch) from the pretreated *Chlamydomonas reinhardtii* microalgae biomass, which is a mixture of starch and glucose accumulated by microalgae grown with CO_2_ [112]. At present, the Calvin-Benson cycle has been reconstructed in *E. coli* [113] and *Pichia pastoris* [114, 115] to use CO_2_ as the only carbon source. Based on the carbon metabolism network and carbon loss advantages of *C. glutamicum*, it is expected that the use of CO_2_ for biological manufacturing by *C. glutamicum* will be a feasible research direction soon.

## Challenges and prospects

*C. glutamicum* provides a flexible protein production technology platform with a relatively complete display, transport, metabolism, and secretion systems. In addition, many techniques for producing chemicals using sustainable biomass raw materials have been reported through the surface display system of *C. glutamicum*. This shows that the development of the display system will expand the potential applications of surface engineering microorganisms to industrial applications. Using biotechnology, exhibiting and using extracellular enzymes to engineer *C. glutamicum* as a cell refinery in the future is ideal, but challenging. To realize the potential of *C. glutamicum* in cell refineries in the future, it is necessary to further optimize the display system of *C. glutamicum*, including the following aspects: (1)The display system of *Corynebacterium* requires optimization to improve the efficiency. Bacterial protein displays have not been widely used in industrial environments, partly because it limits the number and stability of display proteins [116]. (2) Improve the expression level of foreign proteins, optimize the transportation and coordination of carbon metabolism and nitrogen metabolism network. The low production efficiency of the recombinant protein of *C. glutamicum* is an urgent problem to be solved in the biorefinery of *C. glutamicum*. The metabolic burden is a phenomenon known in heterologous expression systems. It is due to the overexpression of foreign proteins occupying a large part of the intracellular nutrient flux, which affects the balance of cell metabolism [49]. (3) Improve the study of metabolic regulation mechanisms. *C. glutamicum* still lacks an in-depth understanding of the basic regulatory principles for the synthesis of central metabolic enzymes under different environmental conditions and their effects on cell growth [117]. In recent years, research on the catalysis and regulation mechanism [118–121] and resistance of aromatic compounds [122], genome simplification, optimization of gene editing tools [123, 124], and research on ribosomal switches [125] and biosensors [126] have gradually increased. With the developments in metabolic engineering, systems biology, and synthetic biology, *C. glutamicum* will hopefully become a promising and advantageous biorefinery factory.

## Data Availability

Not applicable.
